# Reply to Discussion by R. A. Henderson of ‘Kinematics of the Clarke River Shear Zone (northeastern Australia) and implications for the tectonic evolution of the Tasmanides’

**DOI:** 10.1080/08120099.2026.2634689

**Published:** 2026-03-26

**Authors:** R. T. Rodrigues, G. Rosenbaum

**Affiliations:** School of the Environment, The University of Queensland, Brisbane, QLD, Australia

## Introduction

We welcome the opportunity to address R. A. Henderson’s discussion on Rodrigues *et al*. (2024). Henderson raised several important points concerning: (i) the conditions of emplacement of the Craigie Tonalite; (ii) the effects of the Tabberabberan Orogeny on the Clarke River Shear Zone; (iii) the relationship between reactivation of the Clarke River Shear Zone and development of the Broken River Orocline; (iv) the significance of the earliest sinistral phase along the Clarke River Shear Zone; and (v) the proposed tectonic model, including the role of the Policeman Fault in subduction segmentation.

## Conditions during emplacement of the Craigie Tonalite

One of the points raised by Henderson concerns the conditions of emplacement of the Craigie Tonalite, which is a Late Devonian intrusion (410 ± 1 Ma; Rodrigues *et al*., 2024) belonging to the Pama Igneous Association. In particular, Henderson questioned the interpretation that the Craigie Tonalite was emplaced syn-kinematically at mid- to lower-crustal levels.

**Figure 1.**
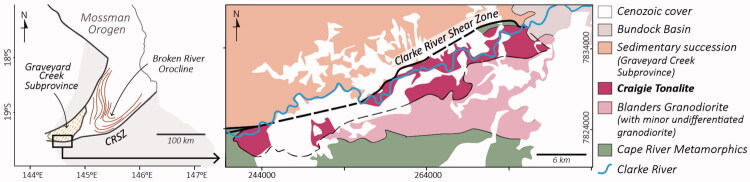
Simplified geological map of the western segment of the Clarke River Shear Zone and adjacent units (after Withnall & Lang, 1993), illustrating the elongated lenticular map-view geometry of the Craigie Tonalite.

The syn-kinematic emplacement of the Craigie Tonalite was inferred based on field observations, including: (i) the parallelism between the tectonic foliation developed within the tonalite and the mylonitic foliation along the Clarke River Shear Zone; and (ii) the lenticular geometry of the intrusion, which is aligned with the structural grain of the shear zone. This lenticular character is clearly expressed in geological maps, which show an elongated map-view geometry of the Craigie Tonalite adjacent to the Clarke River Shear Zone (Figure 1). Both attributes, the lenticular shape of the intrusion and the concordant magmatic and tectonic foliations, are consistent with syn-tectonic emplacement, as documented in natural examples and experimental studies (*e.g.* Bessière *et al*., 2018; Passchier & Trouw, 2005; Vernon *et al*., 1989). We will provide additional data on this issue in a forthcoming paper.

Henderson also questioned the interpretation that the Craigie Tonalite was emplaced at mid- to deep-crustal levels. This interpretation is supported by field and microstructural evidence, including the absence of a distinct contact metamorphic aureole in the Cape River Metamorphics and the presence of gradational contacts between the intrusion and its host rocks (Rodrigues *et al*., 2024). Such observations suggest emplacement under high-temperature ductile conditions (*e.g.* Winter, 2010). Further evidence for emplacement below the brittle–ductile transition under amphibolite-facies conditions is provided by microstructural observations, which show crystal-plastic deformation of amphibole and dynamically recrystallised plagioclase. Such deformation mechanisms are consistent with syn-tectonic emplacement at middle to lower crustal levels (*e.g.* Berger & Stünitz, 1996; Bucher & Frey, 2002).

Henderson’s suggestion that the emplacement of the Craigie Tonalite occurred at shallow crustal levels is based on the presence of a broadly coeval sedimentary succession north of the Clarke River Shear Zone (Graveyard Creek Subprovince; Figure 1). However, this interpretation does not consider differential vertical motions associated with basin development. Considering the evidence for coeval ductile deformation south of the Clarke River Shear Zone and sedimentation north of it, it seems likely that the southern block (Craigie Tonalite) was uplifted relative to the northern block (Graveyard Creek Subprovince). Such spatial decoupling is commonly observed in tectonically active sedimentary basins (*e.g.* DeCelles & Giles, 1996; Korsch & Totterdell, 2009); it is consistent with the evidence of general shear (rather than simple shear) during deformation (Rodrigues *et al*., 2024), and the overall tectonic evolution of the basin that hosts the Graveyard Creek Subprovince (Rodrigues, 2026).

## Effects of the Tabberabberan Orogeny on the Clarke River Shear Zone

Henderson argued that the influence of the Tabberabberan Orogeny on the deformational history of the Clarke River Shear Zone was underestimated by Rodrigues *et al*. (2024). He suggested that this Middle to Late Devonian orogenic event significantly affected rocks of the Broken River Province and may have contributed to reactivation along the Clarke River Shear Zone.

The main contractional phase of the Tabberabberan Orogeny in northeastern Australia occurred during the Late Devonian–early Carboniferous (Henderson & Withnall, 2013). Based on our data, however, this phase of deformation had only limited expression in the Graveyard Creek Subprovince. In this subprovince, the Tabberabberan Orogeny may explain the existence of a Late Devonian low-angle unconformity between the Broken River and Bundock Creek groups (Henderson & Withnall, 2013), but there is little evidence that rocks in the Graveyard Creek Subprovince were deformed during this event. The dominant deformation in the Graveyard Creek Subprovince was younger (Carboniferous), as indicated by the age of syn-kinematic intrusions (*ca* 325 Ma), which were emplaced simultaneously with the development of macroscopic folds in the area (Rosenbaum *et al*., 2024).

The western sector of the Clarke River Shear Zone, investigated by Rodrigues *et al*. (2024), is located immediately adjacent to the Graveyard Creek Subprovince (Figure 1). Accordingly, there is no evidence to suggest that this sector experienced significant shear zone activity during the Late Devonian–early Carboniferous. Nevertheless, the possibility that other sectors of the Clarke River Shear Zone were reactivated during the Tabberabberan Orogeny cannot be ruled out.

## Relationship between reactivation along the Clarke River Shear Zone and formation of the Broken River Orocline

Henderson proposed a potential genetic link between the Clarke River Shear Zone and the orocline (referred to as the Broken River Orocline in Rodrigues *et al*., 2025) (Figure 1). Our field-based structural data from the Broken River Orocline (Rodrigues *et al*., 2025) indicate that oroclinal bending developed predominantly during the Late Devonian (and/or possibly Middle Devonian). The constraints on the timing of oroclinal bending (figure 4 in Rodrigues *et al*., 2025) are based on the observation that Early Devonian units (*e.g.* Kangaroo Hills Formation) are deformed by the orocline, whereas overlying earliest Carboniferous (Tournaisian) units (Clarke River Basin) are unaffected by the orocline. Our geochronological and field-based structural work does not support significant activity along the Clarke River Shear Zone during the Middle–Late Devonian (Rodrigues *et al*., 2024). We therefore think that the suggestion that the orocline formed in response to shearing along the Clarke River Shear Zone is tenuous (but not impossible).

## Significance of the earliest sinistral phase

Henderson raised the possibility that the early sinistral ductile deformation along the Clarke River Shear Zone (recorded in mylonitic rocks of the Cape River Metamorphics) may reflect regional fabrics unrelated to the shear zone (*e.g.* Fergusson *et al*., 2005; Fergusson & Henderson, 2013). While this explanation cannot be ruled out, we maintain that our interpretation is supported by strong evidence.

Sinistral deformation in the Cape River Metamorphics is expressed by mesoscale ductile structures (*e.g.* asymmetric folds and sheared leucosomes) and a steep, subvertical foliation, which is broadly parallel to the shear zone. This parallelism is particularly evident in areas proximal to the Clarke River Shear Zone (see figures 4 and 7 in Rodrigues *et al*., 2024), whereas away from the shear zone, the dominant fabrics display markedly different orientations. The evidence therefore suggests a spatial association between the steep shear-parallel fabrics and the deformation along the Clarke River Shear Zone. Comparable structural relationships have been documented in the eastern sector of the Clarke River Shear Zone, where sinistral shearing has been inferred from the rotation of pre-existing fabrics and interpreted to have occurred during the Late Ordovician and/or early Silurian (Dirks *et al*., 2021).

Additional support for sinistral deformation is provided by aeromagnetic data, which reveal sigmoidal geometries defined by highly magnetised linear anomalies south of the Clarke River Shear Zone (figure 3 in Rodrigues *et al*., 2024). A sinistral interpretation has also been proposed by Abdullah and Rosenbaum (2018) based on regional aeromagnetic analyses. Sinistral kinematic indicators are also locally expressed in the map-view geometry of Silurian units along the northeastern sector of the shear zone (figure 3c in Rodrigues *et al*., 2024).

## Tectonic model and the role of the Policeman Fault in subduction segmentation

Henderson identified potential limitations in the tectonic model proposed by Rodrigues *et al*. (2024), whereby the Policeman Fault was assumed to accommodate differential kinematics associated with subduction segmentation. Henderson noted that the Policeman Fault was identified primarily from aeromagnetic imagery, tentatively interpreted as a sinistral structure (Abdullah & Rosenbaum, 2018), but with no clear field evidence supporting this suggestion. We agree that the kinematics of the Policeman Fault is poorly constrained, and that further investigation is needed to evaluate the three-dimensional geometry and deformation history of this fault.

## Final remarks

We acknowledge the value of this discussion and welcome the opportunity to further evaluate and refine tectonic interpretations of the Clarke River Shear Zone and surrounding areas. The data presented by Rodrigues *et al*. (2024) are based primarily on detailed field observations integrated with structural and microstructural analyses and zircon U–Pb geochronology. Our interpretations of the deformational history of the Clarke River Shear Zone are directly derived from these datasets and represent internally consistent geological observations. The broader tectonic implications are open to reinterpretations, which could be tested when more data become available.

## References

[CIT0001] Abdullah, R., & Rosenbaum, G. (2018). Devonian crustal stretching in the northern Tasmanides (Australia) and implications for oroclinal bending. *Journal of Geophysical Research: Solid Earth*, *123*(8), 7108–7125. 10.1029/2018JB015724

[CIT0002] Berger, A., & Stünitz, H. (1996). Deformation mechanisms and reaction of hornblende: Examples from the Bergell tonalite (Central Alps). *Tectonophysics*, *257*(2-4), 149–174. 10.1016/0040-1951(95)00125-5

[CIT0003] Bessière, E., Rabillard, A., Précigout, J., Arbaret, L., Jolivet, L., Augier, R., Menant, A., & Mansard, N. (2018). Strain localization within a syntectonic intrusion in a back-arc extensional context: The naxos Monzogranite (Greece). *Tectonics*, *37*(2), 558–587. 10.1002/2017tc004801

[CIT0004] Bucher, K., & Frey, M. (2002). *Petrogenesis of metamorphic rocks* (8th ed.). Springer. 10.1007/978-3-540-74169-5

[CIT0005] DeCelles, P. G., & Giles, K. A. (1996). Foreland basin systems. *Basin Research*, *8*(2), 105–123. 10.1046/j.1365-2117.1996.01491.x

[CIT0006] Dirks, H. N., Sanislav, I. V., & Abu Sharib, A. S. A. A. (2021). Continuous convergence along the paleo-Pacific margin of Australia during the early Paleozoic: Insights from the Running River Metamorphics, NE Queensland. *Lithos*, *398-399*, 106343. 10.1016/j.lithos.2021.106343

[CIT0007] Fergusson, C. L., & Henderson, R. A. (2013). Thomson Orogen. In P. A. Jell (Ed.), *Geology of Queensland* (pp. 113–221). Geological Survay of Queensland.

[CIT0008] Fergusson, C. L., Henderson, R. A., Lewthwaite, K. J., Phillips, D., & Withnall, I. W. (2005). Structure of the Early Palaeozoic Cape River Metamorphics, Tasmanides of north Queensland: Evaluation of the roles of convergent and extensional tectonics. *Australian Journal of Earth Sciences*, *52*(2), 261–277. 10.1080/08120090500139372

[CIT0009] Henderson, R. A., & Withnall, I. W. (2013). Broken River Province. In P. A. Jell (Ed.), *Geology of Queensland* (pp. 250–280). Geological Survey of Queensland.

[CIT0010] Korsch, R. J., & Totterdell, J. M. (2009). Evolution of the Bowen, Gunnedah and Surat Basins, eastern Australia. *Australian Journal of Earth Sciences*, *56*(3), 271–272. http://doi: 10.1080/08120090802695733

[CIT0011] Passchier, C. W., & Trouw, R. A. J. (2005). *Microtectonics* (2nd ed.). Springer.

[CIT0012] Rodrigues, R. T. (2026). The role of crustal-scale structures on the evolution of orogenic curvatures in northeastern Australia: Implications for the Palaeozoic evolution of eastern Gondwana [unpublished PhD thesis]. The University of Queensland.

[CIT0013] Rodrigues, R. T., Rosenbaum, G., & Heaslop, R. (2025). Anatomy of a curved orogen: The Broken River Orocline in the northeastern Tasmanides (Australia). *Tectonics*, *44*(8), e2025TC008908. 10.1029/2025TC008908

[CIT0014] Rodrigues, R. T., Rosenbaum, G., & Abdullah, R. (2024). Kinematics of the Clarke River Shear Zone (northeastern Australia) and implications for the tectonic evolution of the Tasmanides. *Australian Journal of Earth Sciences*, *71*(4), 492–512. 10.1080/08120099.2024.2339391 PMC13131164 42077363

[CIT0015] Rosenbaum, G., Barrett, A., Rodrigues, R. T., Allen, C. M., & Weinberg, R. F. (2024). Formation of a dome-and-basin fold interference pattern by granite intrusions. *Geological Society of America Bulletin*, *136*(7-8), 3391–3404. 10.1130/B37297.1

[CIT0016] Vernon, R. H., Paterson, S. R., & Geary, E. E. (1989). Evidence for syntectonic intrusion of plutons in the Bear Mountains Fault Zone, California. *Geology*, *17*(8), 723–726. 10.1130/0091-7613(1989)017<0723:EFSIOP>2.3.CO;2.

[CIT0017] Winter, J. D. (2010). *Principles of igneous and metamorphic petrology* (2nd ed.). Prentice Hall.

[CIT0018] Withnall, I. W., & Lang, S. C. (1993). *Geology of the Broken River Province, North Queensland*. Department of Minerals and Energy, Queensland.

